# Quantitative evaluation of maxillary bone deformation by computed tomography in patients with leprosy

**DOI:** 10.1371/journal.pntd.0006341

**Published:** 2018-03-09

**Authors:** Norio Kasai, Osamu Kondo, Koichi Suzuki, Yoshinori Aoki, Norihisa Ishii, Masamichi Goto

**Affiliations:** 1 Department of Otolaryngology, National Sanatorium Oku-Komyoen, Setouchi City, Okayama, Japan; 2 Department of Otolaryngology, National Sanatorium Nagashima-Aiseien, Setouchi City, Okayama, Japan; 3 Department of Biological Sciences, Graduate School of Science, The University of Tokyo, Bunkyo-ku, Tokyo, Japan; 4 Department of Clinical Laboratory Science, Faculty of Medical Technology, Teikyo University, Itabashi-ku, Tokyo, Japan; 5 Leprosy Research Center, National Institute of Infectious Diseases, Higashimurayama, Tokyo, Japan; 6 National Sanatorium Hoshizuka-Keiaien, Kanoya, Kagoshima, Japan; University of California San Diego School of Medicine, UNITED STATES

## Abstract

**Background:**

Facial deformation as a sequela of leprosy is caused not only by a saddle nose but also by regression of the maxilla, as well documented in paleopathological observations of excavated skeletal remains of patients with leprosy. However, maxillary changes in living patients have been evaluated only by the subjective visual grading. Here, we attempted to evaluate maxillary bone deformation in patients with leprosy using three-dimensional computed tomography (3D-CT).

**Methods:**

Three-dimensional images centered on the maxilla were reconstructed using multiplanar reconstruction methods in former patients with leprosy (n = 10) and control subjects (n = 5); the anterior-posterior length of the maxilla (M_A-P_) was then measured. The difference between the M_A-P_ of the patients and those of controls was evaluated after compensating for individual skull size. These findings were also compared with those from previous paleopathological studies.

**Findings:**

Three former patients with lepromatous leprosy showed marked atrophy of the maxilla at the prosthion (-8.6, -11.1 and -17.9 mm) which corresponded with the visual appearance of the maxillary deformity, and these results were consistent with paleopathological findings of excavated skeletal remains. Additionally, the precise bone defects of the maxilla could be individually calculated for accurate reconstructive surgery.

**Interpretation:**

We have successfully illustrated maxillary bone deformities in living patients with leprosy. This study also confirmed the maxillary regression described in paleopathological studies.

## Author summary

The sequelae of leprosy are diverse and include facial deformity. A characteristic facial deformity is saddle nose, and the common clinical approach to this facial deformity has been rhinoplasty. Paleopathological studies from excavated skeletal remains have revealed that leprosy also causes deformation of the maxillary bone, which supports the external nose; however, these changes have not been evaluated in living patients with leprosy. In this study, we reconstructed three-dimensional and stereoscopic images of the cranium from CT scan data and evaluated maxillary deformities both qualitatively and quantitatively. Out of the ten former patients with leprosy, three had severe deformity of the maxilla including: atrophy of the anterior nasal spine, resorption of the medial part of the alveolar process, loss of sharpness of the piriform aperture margins, and atrophy of the nasal turbinate and the septum. These changes were consistent with previous findings from paleopathological studies. We also developed a novel method for measuring the anterior-posterior length of the maxilla using 3D-CT images, with compensation for individual differences in cranial size. The calculated values are indicative of the severity of the maxillary defects as well as the thickness of bone that needs to be reconstructed to correct the deformed maxilla.

## Introduction

Leprosy, also known as Hansen’s disease, is a chronic infectious disease caused by *Mycobacterium leprae* (*M*. *leprae*), and classified by Ridley-Jopling’s five-group system [1], from lepromatous (LL) to tuberculoid (TT). Briefly, LL cases show no cellular immunity against *M*. *leprae* and manifest as a generalized disease including nasal cavities that resemble miliary tuberculosis, while TT cases shows cellular immunity and limited disease manifestation resembling the primary complex of tuberculosis [1]. Together with deformities of the hands and feet, facial deformation is one of the serious sequelae of leprosy because these can lead to segregation and prejudice, and often cause difficulties in the social rehabilitation of cured patients. Maxillary lesions in skulls excavated from old leprosarium cemeteries were first described and termed facies leprosa by Moller-Christensen [2]. Later, Andersen et al. [3] described the manifestation of these lesions as rhinomaxillary syndrome. Moreover, the DNA of *M*. *leprae* could be identified in these bones [4–6]. These leprosy-induced maxillary bone deformations have only been visually graded, and objective or morphometric evaluation of the lesions has yet to be performed.

The maxilla supports the external nose structures, and morphological changes in the maxilla greatly affect the shape of the nose and face. However, plastic surgery has only been performed for the correction of saddle nose as an augmentation rhinoplasty (e.g., prosthesis insertion into the nasal dorsum) [7, 8], and almost nothing has been attempted for the repair of the deformed maxilla itself. As a result of this limited reconstruction, persons affected with leprosy often show recurrent saddle nose long after the augmentation rhinoplasty. Thus, there is a clear need to evaluate the precise morphological changes in the maxilla in order to develop fundamental corrective treatments for facies leprosa or rhinomaxillary syndrome.

In this study, we reconstructed three-dimensional CT images of the maxillary bone in patients with a history of leprosy and in control subjects using the volume rendering (VR) and multiplanar reconstruction (MPR). These CT findings were compared with maxillary deformities described in paleopathological studies. Furthermore, after standardization and comparison of leprotic and control data, we succeeded in quantifying the thickness of the maxillary bone defects that require reconstructive surgery.

## Materials and methods

### Patients and data collection

The leprosy group (group L) consisted of patients who had leprosy and were successfully treated with antibiotics (former patients with leprosy). They were all residents of the National Sanatorium Oku-Komyoen and Nagashima-Aiseien, Okayama, Japan in 2015 and had a CT scan involving the maxilla performed within the past 5 years. Thirteen subjects were initially enrolled; however, the quality of CT data was not sufficient in three subjects, so a total of ten subjects were included in the final study. Ages ranged from 68 to 97 years (median, 85.0 years; average, 83.8 years), and the male/female ratio was 7/3. Clinical information was also collected from their medical records, including the type of leprosy, age of onset and duration of the disease. The control group (group C) consisted of adult Japanese people with no history of leprosy that had been examined by CT scan involving the maxilla within the past 2 years. These subjects included 5 participants, four males and one female, ranging in age from 36 to 91 years (median, 65.0 years; average, 62.0 years).

### Measurement of the maxilla from CT images

#### Image reconstruction and comparison

Whole-body CT scanners (Aquilion CX or Activion 16; Toshiba Medical Systems Corp., Tochigi, Japan) were used at the following settings: 120 kV, 100 mAs and 0.5 mm pitch. The volumetric data from the CT images involving the maxilla, with 0.5 mm slice thickness and interval, were imported to the DICOM viewing software “VirtualPlace Fuujin ver. 3.1” (AZE corporation, Tokyo, Japan) for reconstructing the 3D images. The volume of interest (VOI) was limited to the maxillofacial area. The stereoscopic images and images from three planes (axial, coronal and sagittal views) were prepared using the volume rendering (VR) and multiplanar reconstruction (MPR), respectively. The CT window levels (between 200 and 800 HU) and window widths (2000 and 3000 HU) were set and adjusted using the signal/noise ratio.

Three-dimensional CT images were rendered and evaluated in the leprosy and control groups based on the characteristic maxillary bone deformities described for excavated skeletal remnants from graves of leprosy sanatoria and living patients [2–4, 9–15]. These deformities included: (1) atrophy of the anterior nasal spine, (2) resorption of the medial part of the alveolar process, (3) loss of sharpness of the piriform aperture margins, and (4) atrophy of the nasal turbinate and the septum. The presence of saddle nose caused by leprosy was also evaluated. The findings were classified into either two categories, negative (-) and positive (+); or into three categories, negative (-), mild to moderately positive (+), and severely positive (++).

#### Anatomical landmarks and establishment of standard planes

The following cranial landmarks were identified on the reconstructed CT images: porion *(‘po’*, the superior-most lateral point on the margin of the external auditory meatus), orbitale *(‘or’*, the inferior-most point on the margin of the orbit), alveolon (*‘alv’*, the point where the midline of the palate is intersected by a straight tangent connecting the posterior borders of the alveolar crests), nasion (*‘n’*, the point of intersection of the nasofrontal suture and the mid-sagittal plane), prosthion (*‘pr’*, the most anterior point on the alveolar border of the maxilla), and basion (*‘ba’*, the point where the anterior margin of the foramen magnum is intersected by the mid-sagittal plane).

**Fig 1:**
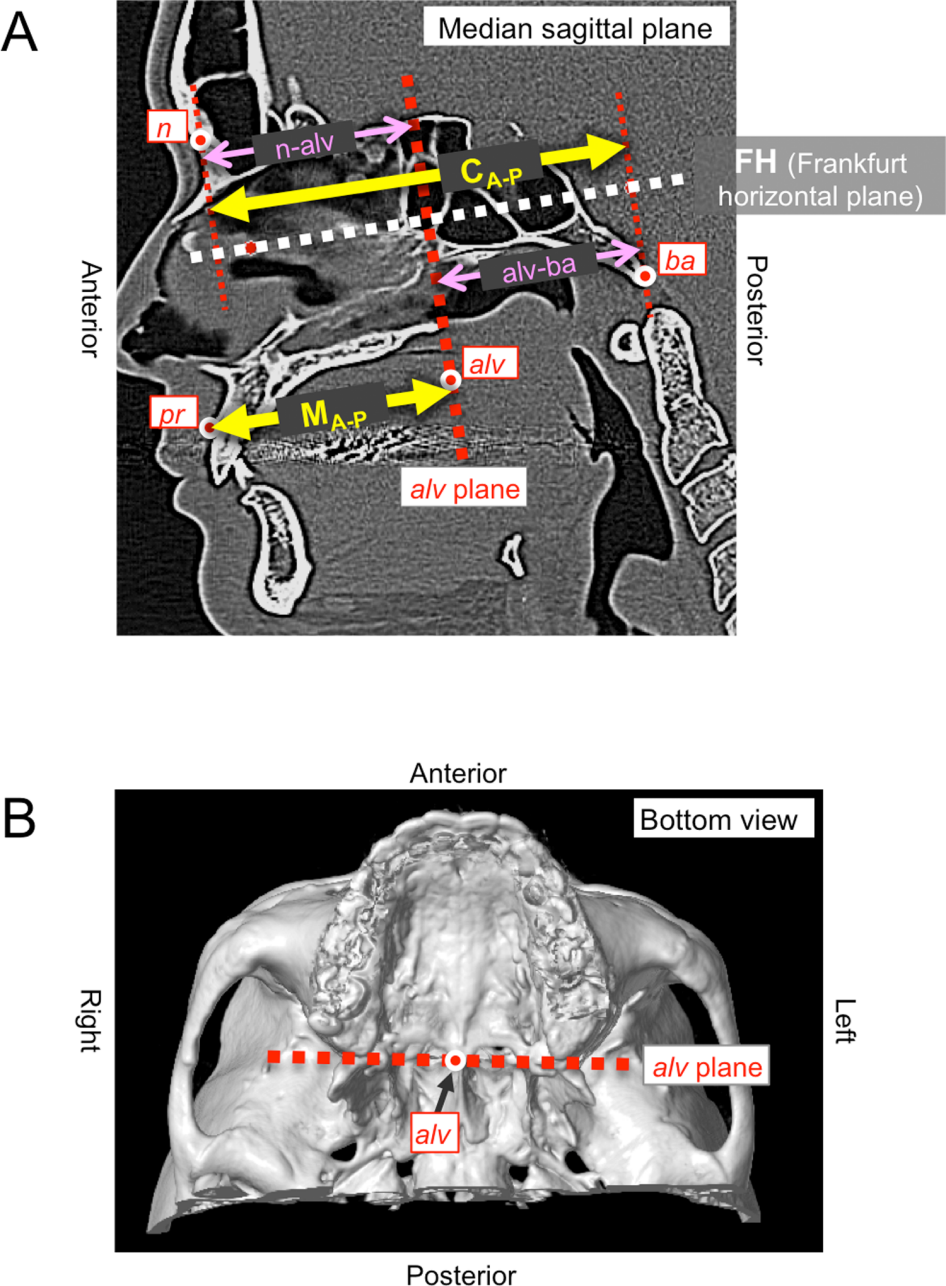
Reference points and planes, and measurement lines on the CT images. A. Measurement lines on the median sagittal plane of the cranium. The volume of interest (VOI) was limited to the maxillofacial area and the anatomical landmarks are identified. The sum of the distance from ‘*n’* to the *‘alv* plane*’* (“*n-alv* distance”) and from the *‘alv* plane*’* to *‘ba’* (“*alv-ba* distance”) was defined as a reference of the cranial anterior-posterior length, termed “C_A-P_”. The distance from *‘pr’* to the *‘alv* plane*’* was defined as the anterior-posterior length of the maxilla, termed “M_A-P_”. *‘alv’*: the point where the midline of the palate is intersected by a straight tangent connecting the posterior borders of the alveolar crests. *‘n’*: the point of intersection of the nasofrontal suture and the mid-sagittal plane. *‘pr’*: the most anterior point on the alveolar border of the maxilla. *‘ba’*: the point where the anterior margin of the foramen magnum is intersected by the mid-sagittal plane. B. Definition of ‘*alv’* plane. The coronal plane which is orthogonal to the FH (Frankfurt horizontal plane) and parallel to the line connecting the left and right *‘po’*, and passes through *‘alv’*, was defined as the *‘alv* plane*’*.

The Frankfurt horizontal plane (FH), which passes through the left *‘or’* and right and left *‘po’*, was set as a reference plane (Fig 1A). The coronal plane was defined as orthogonal to the FH and parallel to a line connecting both *‘po’*. The plane parallel to the coronal plane and passing through *‘alv’* was termed the *‘alv* plane*’*, which defines the posterior border of the maxillofacial portion of the cranium (Fig 1B). All measurement lines were drawn on the median sagittal plane and parallel to the FH.

#### Measurement of the anterior-posterior length of the cranium (C_A-P_) and maxilla (M_A-P_)

The distances from *‘n’* to the *‘alv* plane*’* and from the *‘alv* plane*’* to *‘ba’* were termed the “*n-alv* distance” and “*alv-ba* distance”, respectively (Fig 1A). The sum of these two values was defined as a reference of the cranial anterior-posterior length, termed “C_A-P_”. The distance from *‘pr’* to the *‘alv* plane*’* was defined as the anterior-posterior length of the maxilla, termed “M_A-P_”. All measurements were repeated at least three times, and the average value was used.

#### Comparison of C_A-P_ and M_A-P_

The M_A-P_/C_A-P_ ratio was calculated to adjust for individual differences in cranial size, enabling a direct comparison between cases. Additionally, the average M_A-P_/C_A-P_ ratio of group C was set as a tentative control value, and the M_A-P_/C_A-P_ value from each group L subject was compared to this control value. The difference indicates the severity of the maxillary defect, and also indicates the thickness of the bone in need of reconstruction.

### Ethical considerations

This research was approved by the local ethics committees of Oku-Komyoen (approval #27–11) and Nagashima-Aiseien (#27–20). In accordance with the WMA Declaration of Helsinki (1975, revised 2008), a detailed explanation was provided to the subjects in advance. Only subjects who consented to this research by signing the informed consent form were included in the study.

## Results

The clinical manifestations of group L (10 cases) were classified as eight lepromatous (LL), one tuberculoid (TT), and one borderline tuberculoid (BT; Table 1). The maxillary bone deformities from all subjects could easily be evaluated by 3D-CT imaging. Atrophy of the anterior nasal spine was observed in four of the LL cases (ID 5, 9, 11, and 12), and resorption of the medial part of the alveolar process was also found in these four cases. Loss of sharpness of the piriform aperture margins was detected in five of the LL cases (ID 2, 5, 9, 11, and 12), and atrophy of the nasal turbinate and septum was seen in six and five of the LL cases, respectively. Subjects with two or more deformities also had saddle nose. No deformities were observed in the TT and BT cases (Table 1).

**Table 1:** Evaluation of maxillary deformities from 3D-CT images.

Group	ID	Age	Gender	History of leprosy				Visual evaluation of deformity					Saddle nose	Measured and calculated deformity values				
				Type	Age of onset	Duration from onset to initiation of chemotherapy (years)	Duration from initiation of chemotherapy to negative skin smear (years)	Atrophy of anterior nasal spine	Resorption of alveolar process	Loss of sharpness of piriform aperture	Atrophy of nasal turbinate	Perforation of nasal septum		Measured M_A-P_^*1^ (mm)	Measured C_A-P_^*2^ (mm)	M_A-P_/C_A-P_	Estimated M_A-P_^*3^ (mm)	Maxillary defect^*4^ (mm)
L (leprosy)	1	77	F	LL	10	4	13	-	-	-	+	-	-	42.45	79.50	0.53	45.40	-2.94
	2	79	F	LL	10	5	23	-	-	+	-	-	-	51.03	85.83	0.59	49.01	2.02
	4	82	M	LL	10	9	14	-	-	-	+	+	+	45.73	85.70	0.53	48.94	-3.21
	5	88	M	LL	12	13	29	++	++	+	++	+	++	44.05	92.19	0.48	52.64	-8.60
	6	68	F	LL	12	5	9	-	-	-	-	-	-	50.55	84.20	0.60	48.08	2.47
	7	75	M	BT	14	5	11	-	-	-	-	-	-	51.73	95.85	0.54	54.73	-3.01
	8	97	M	TT	10	unknown	unknown	-	-	-	-	-	-	51.83	90.42	0.57	51.63	0.20
	9	90	M	LL	12	15	16	++	++	+	++	+	++	31.28	86.20	0.36	49.22	-17.94
	11	94	M	LL	22	5	unknown	+	+	+	++	+	++	53.50	100.33	0.53	57.29	-3.79
	12	88	M	LL	13	11	13	++	++	+	++	+	++	41.00	93.03	0.44	53.12	-12.12
	mean	83.8			12.5	8.0	16.0							46.32	89.33	0.52	51.00	-4.69
	SD	9.1			3.6	4.1	6.7							6.88	6.19	0.07	3.53	6.46
C (control)	C1	41	M					-	-	-	-	-	-	55.45	98.23	0.56	56.09	-0.64
	C2	77	M					-	-	-	-	-	-	51.51	86.52	0.60	49.40	2.11
	C3	65	M					-	-	-	-	-	-	53.17	93.69	0.57	53.50	-0.32
	C4	91	M					-	-	-	-	-	-	47.93	87.68	0.55	50.06	-2.14
	C5	36	F					-	-	-	-	-	-	51.60	88.50	0.58	50.53	1.07
	mean	62.0												51.93	90.92	0.57	51.92	0.01
	SD	23.4												2.75	4.92	0.02	2.81	1.63

Each value was measured at least three times and the average value is shown.

*1: Anterior-posterior length of the maxilla (distance between prosthion and the alveolon plane) measured from 3D-CT images.

*2: Anterior-posterior length of the cranium (distance between nasion and basion) measured from 3D-CT images.

*3: Each C_A-P_ value was multiplied by 0.57 (mean M_A-P_/C_A-P_ of group C).

*4: Estimated M_A-P_ was subtracted from the measured M_A-P_.

**Fig 2:**
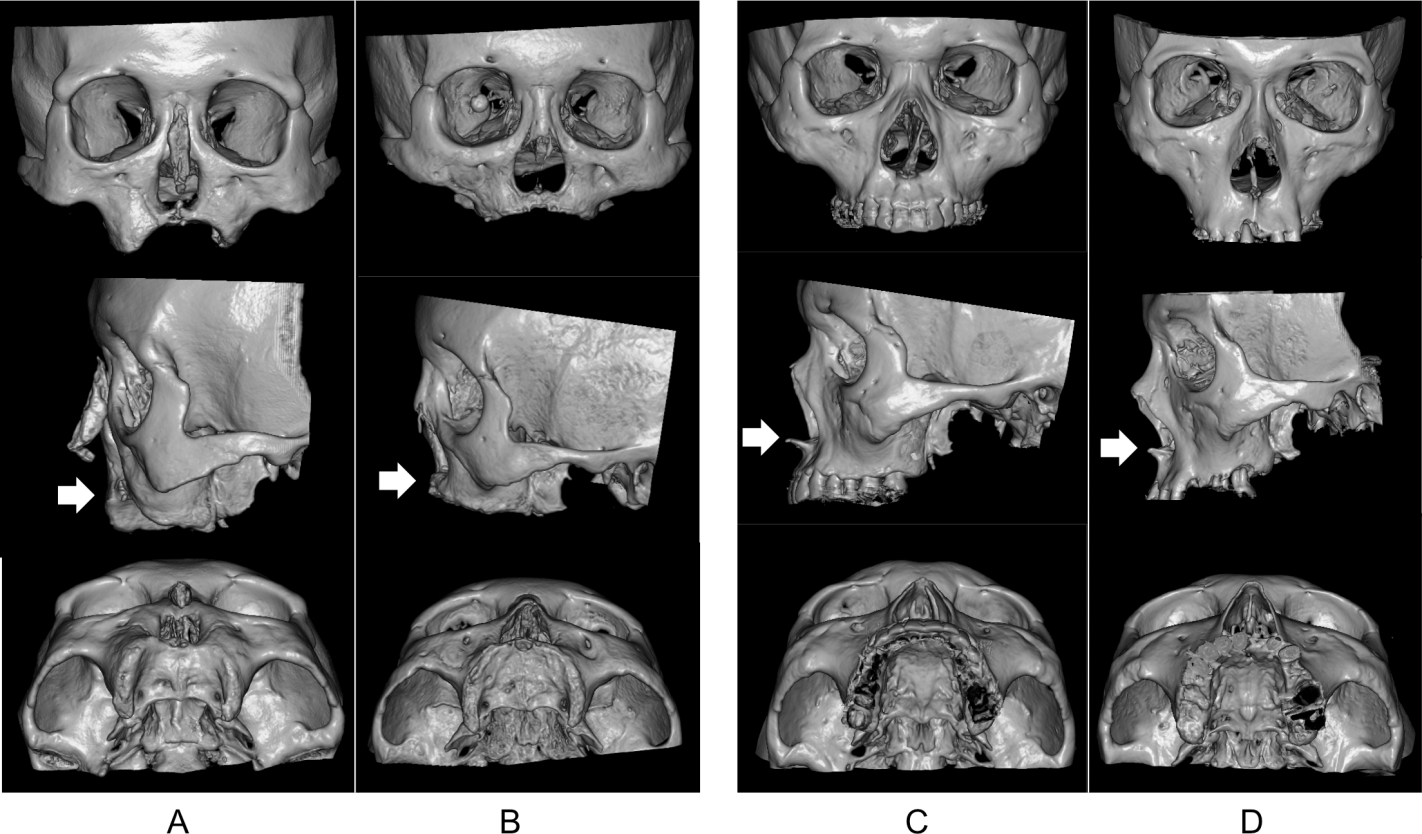
Reconstructed stereoscopic CT images of the maxilla. Two severely deformed cases, A (ID 9) and B (ID12), and two control cases, C (ID C1) and D (ID C3), are shown. The front, left side, and bottom view of the cranium are shown from top to bottom. The deformities of the anterior nasal spine and the alveolar process are easily observed in A and B. The result is that the retracted surface of the central maxilla and the regression of the alveolar process look nearly symmetric and crescentic. Arrows indicate the anterior nasal spine (ANS) or the corresponding point.

Former leprosy patients with deformities of the anterior nasal spine and the alveolar process showed retraction of the surface of the central maxilla, and regression of the alveolar process that looked nearly symmetric and crescentic in severe cases (ID 5, 9 and 12; Fig 2). These features suggest that the deformity of the anterior nasal spine and the alveolar process extended in two directions, posteriorly and superiorly. The lost portion of the maxillary bone seems to correspond to the premaxilla or the incisive bone, since the incisive canal located at the boundary between the premaxilla and maxilla was nearly exposed on the surface of the maxilla or completely lost in severe cases (ID 5, 9 and 12; Fig 2). These findings were consistent with previous paleopathological studies of excavated skulls and with X-ray studies in living patients with leprosy [2–4, 9–15].

The severity of deformation differed among subjects (Table 1). Although all severe cases (ID 5, 9 and 12) were of the LL type, other LL cases showed very minimal changes to the maxilla. Interestingly, the cases with severe deformity of the alveolar process and anterior nasal spine also had advanced atrophy of both the intranasal structures and the margin of the piriform aperture. The three most severe cases (ID 5, 9 and 12) experienced a longer duration from the disease onset to the initiation of chemotherapy (13, 15 and 11 years, respectively).

The average M_A-P_/C_A-P_ ratio of group C (n = 5) was 0.57 ± 0.02, and the variation was small with a minimum value of 0.55 and a maximum value of 0.60. In contrast, the average M_A-P_/C_A-P_ ratio of group L (n = 10) was 0.52 ± 0.07. The smaller average and the larger standard deviation were mostly due to the small M_A-P_/C_A-P_ ratios in subjects ID 5, 9 and 12 (0.48, 0.36 and 0.44, respectively). Thus, calculations of the M_A-P_/C_A-P_ ratio could represent a universal parameter that enables the evaluation of deformities among individuals with different cranial sizes.

From the average value of group C, a tentative reference value for the M_A-P_/C_A-P_ ratio was set at 0.57. In order to calculate the missing thickness of the maxilla, each C_A-P_ value from group L was multiplied by 0.57 to show the estimated value of the M_A-P_, and the measured value of the M_A-P_ was subtracted. The calculated defect size in group L ranged from +2.5 mm to -17.9 mm, and the severe cases (ID 5, 9 and 12) showed remarkably large values (-8.6 mm, -17.9 mm and -12.1 mm, respectively). These values could be utilized to estimate the size of an implant, autologous bone or prosthesis for reconstruction of the deformed maxilla.

## Discussion

The maxillary deformities in patients with leprosy are collectively termed facies leprosa [2] or rhinomaxillary syndrome [3]. The characteristic findings of the maxilla in cases with leprosy are summarized as follows: the anterior nasal spine shows progressive resorption and total loss at the end of the process, the resorption of the alveolar process leads the incisors to fall out and become crescentic in profile, the margins of nasal aperture lose the sharpness symmetrically, the palatine process shows bone erosion and perforation, and intranasal structures especially the turbinates and septum result in atrophy and perforation [3]. Recent reports [4, 14, 15] also showed similar findings from excavated skeletal remains. In 2001, Boldsen [16] tried to estimate the frequency of leprosy from hundreds of skeletal samples using a two-grade scoring system for these maxillary changes. Results identified a high sensitivity/specificity of these findings for the evaluation of leprosy and a high prevalence of leprosy in samples from cemeteries associated with leprosy hospitals. Given that *M*. *leprae* DNA has been detected in excavated skeletal remains within maxillary bones with these deformities, it is evident that these defects are associated with leprosy [4–6]. Thus, these reports have identified that the characteristic changes in excavated maxilla are likely caused by leprosy.

Several studies attempting to evaluate maxillary deformities in living patients have performed regular palpation or lateral X-ray, or vertical measurement of the alveolar process by dental X-ray [9, 11–13]. However, it is difficult to make quantitative evaluations using these conventional methods. The recent evolution of CT scanning and image processing technology have made it possible to reconstruct three-dimensional images of organs as well as to obtain exact measurements in any plane. Therefore, we attempted to apply this technology to patients with leprosy to evaluate maxillary bone deformities both qualitatively and quantitatively.

The use of stereoscopic 3D-CT images enabled easier qualitative evaluation since the images could be observed in any direction. Results indicated that the anterior nasal spine and alveolar process were clearly deformed in four LL cases of the ten former patients. These findings were in accordance with previous observations from excavated leprotic skeletal remains, and also confirmed that the observed changes in these historical specimens primarily occurred while they were still alive, rather than resulting from artificial modification that occurred during the long period in which they were buried under ground.

Although the reason why the median part of the maxilla is intensively destroyed has not been elucidated, Rendall et al. [17] found relatively low temperatures in the premaxillary region and suggested that cold blood flow from the heavily infected nasal cavity enabled the bacilli to spread into this area. Anatomically, the premaxilla is initially separated but later fuses with the maxilla during embryonic stages [18], possibly resulting in poor blood supply and susceptibility to ischemia. These suggest the frontocentral part of maxilla might be one of the “hot spot” of the infectious lesions of leprosy, as lesions of *M*. *leprae* infection appear on relatively cool area of patients. As a result, it is supposed that premaxilla is directly infiltrated and destroyed by infection, and/or leproma in the nasal cavity compresses the vessel and cause necrosis of this bone.

In the general population, many aged subjects show atrophy of the maxillary alveolar processes both horizontally and vertically, resulting in low and smooth protuberance [19]. This is because the loss of their teeth, and its major cause is thought to be chronic infection and inflammation due to periodontal disease. On the other hand, the deformities found in the leprosy cases in our study showed the median frontal part was dominantly affected and the characteristic U-shape of the process was interrupted. Møller-Christensen [11] reported a follow up study of a LL case who showed that upper incisors became loose and were finally lost while other teeth were preserved. Matos and Santos [20] inspected an excavated maxilla, whose age was estimated to have been between 13 and 19 years old at the time of death, and reported that it showed the rhinomaxillary syndrome and alveoli of the upper incisors were almost completely reabsorbed, though the molars and premolars still remained on the alveolar process. These suggest the alveolar change in leprosy is different from the aging changes in morphology.

It is well documented that facial deformities and intranasal changes are mainly observed in lepromatous type [13]. Michman and Sagher [9] found atrophy of anterior nasal spine and alveolar process in 20/30 (67%) of L, 3/10 (30%) of I, and 1/4 (25%) of T, indicating that the abnormalities were not confined but more frequent is L type. Suenaga et al. [21] evaluated the pantomography of 706 cases in a leprosy sanatorium in Japan, and found the bone atrophy of maxillary anterior alveolar process to be 20% in L, 7% in B, and 8% in T. Taheri et al. [22] reported that atrophy of anterior nasal spine in 14/46 (30%) of LL and 0/19 (0%) of TT. These overall data indicate that maxillary changes are more frequent in the lepromatous form than in the tuberculoid form. Nah et al. [13] showed that atrophy of the maxillary bone was more frequent in LL cases, and increased in severity with longer duration of the disease. Other studies have shown that cases with severe atrophy of the maxilla suffered from saddle nose and perforation of the nasal septum [9, 12]. In our study, severe maxillary deformities were seen in three cases, all of which were of the LL type and showed both perforation of the nasal septum and saddle nose. In particular, the most severely deformed case (ID 9; Fig 2A) had completely lost the nasal septum and nasal cavity resulting in a single, empty space.

Conversely, four other LL patients in the present study appeared to have no regression of the maxilla. To identify the cause of this difference, we focused on the history of the disease. As in a previous report [13], the duration from disease onset to initiation of chemotherapy was substantially longer, more than 10 years, in the three most severe cases. However, we could not find any differences in the age of onset or the duration to a negative skin smear. These findings suggest the importance of early detection and early initiation of treatment for the prevention of maxillary deformities.

In this study, we measured the distances between cranial anatomical landmarks on 3D-CT images, and compared the results between patients previously treated for leprosy and control subjects. This method is quantitative and quite different from classical approaches in osteoarcheology or paleopathology which involve the direct measurement of actual bone samples and cannot be applied to living subjects. Until now, CT scan has primarily been used to evaluate mucosal lesions of the nasal cavity or paranasal sinuses in leprosy patients [23, 24] and, to date, there have been no reports on the measurement or quantitative evaluation of the maxillary bone by CT.

Given that the maxilla is part of the cranium, individual differences in cranial size must be accounted for when comparing results among cases. In this study, we first measured the anterior-posterior length of the cranium using several anatomical landmarks, and defined this as the cranial reference length, C_A-P_. We then calculated the M_A-P_/C_A-P_ ratios and performed comparisons between cases. The M_A-P_/C_A-P_ ratios in group C ranged between 0.55 and 0.60, and the variation was very small despite differences in age and gender. Moreover, in cases with no maxillary deformities based on qualitative evaluation in group L, the ratio was similar (0.53–0.60) to that of group C. It might be possible that the average value of the M_A-P_/C_A-P_ ratio is between 0.55 to 0.60 for the most Japanese populations, though much more control samples must be evaluated. In contrast, the three cases in group L with severe deformities of the maxilla had an average ratio of less than 0.48; the most severe case was 0.36. Since these values were consistent with the qualitative severity of the maxillary deformations, we consider that the M_A-P_/C_A-P_ ratio could be one of the helpful parameters for determining the deformity of the maxilla and is suitable for comparative studies among a variety of cases. Moreover, the difference between the estimated M_A-P_ and measured M_A-P_ indicates the atrophied thickness of the maxilla, and this value corresponds to the thickness of the bone or prosthesis that is needed to accurately reconstruct the deformity. Thus, this method may become a tool for reconstructive surgery of maxillary defects caused by leprosy.

As a cautionary point in this study, we should draw attention to the small sample sizes of leprosy and control cases. The whole population of leprosy (cured residents) in Oku-Komyoen was about 130, and their ratio of the type of leprosy was LL = 63%, BL = 5%, BT = 16%, TT = 8%, uncertain = 8%. We could retrieve 13 CT images fulfilling the conditions from this population as the candidates of group L, though we had to remove three cases because of narrow scanning area or inadequate resolution of the CT data. As a result, the 10 cases remained for group L. Additionally, it was also quite difficult to increase the control cases in group C. CTs were taken in patients with no history of leprosy for the evaluation of rhinosinusitis or sinus tumor in the same institute, but the number of patients was quite small because the sanatorium is located in a small island. In future, by further applying this method in more patients and controls in other sanatoria or other countries, it will be possible to evaluate the gender variation and racial differences as well as rendering adequate cutoff values for the clinical application.

In conclusion, although further analysis using a larger number of samples is needed, our methodology using 3D-CT represents a promising tool for the quantitative evaluation of maxillary deformities associated with leprosy and other maxillary lesions. This approach may also contribute to novel surgical treatments for facial deformity, and eliminate the social segregation and prejudice attributed to leprosy.
